# EHMTI-0292. The effect of body fat mass and fat free mass on migraine headache

**DOI:** 10.1186/1129-2377-15-S1-E29

**Published:** 2014-09-18

**Authors:** S Razeghi jahromi, M Abolhasani, A Meysamie, M Togha

**Affiliations:** 1 Multiple Sclerosis Research Center-Neuroscience Institute, Tehran University of Medical Sciences, Tehran, Iran; 2 Department of Community and Preventive Medicine, Tehran University of Medical Sciences, Tehran, Iran; 3 Iranian Center of Neurological Research-Neuroscience Institute, Tehran University of Medical Sciences, Tehran, Iran

## Introduction

Obesity seems to be associated to migraine headache. Increase in body fat, especially ingluteofemoral region, elevates adiponectin and leptin secretion which in turn impair inflammatory processes that could be contributing to migraine risk.

## Aims

This study was designed to assess the relationship between body composition and risk of migraine.

## Methods

In this cross-sectional study, 1510 middle-aged women who were visited in a weight reduction clinic of university were recruited. Migraine was diagnosed with HIS criteria. Body composition parameters including total fat mass (FATM), total fat free mass (FFM), truncal fat mass (TFATM), and truncal fat free mass (TFFM) was assessed using bioelectric impedance. We further assessed cardiovascular risk factors and smoking as confounding factors. To determine the real association between different variables and risk of migraine, the associations were adjusted by multivariate logistic regression analysis.

## Results

Elevation in fasting blood sugar, total cholesterol, LDL cholesterol, FFM, TFFM, and waist-to-hip ratio increased the risk of migraine. When the associations were adjusted for other factors, only the association between migraine and FFM remained statistically significant.

## Conclusion

Lower FFM increased the risk of migraine in overweight and obese individuals. In the other words, higher fat free mass could be a protective factor for migraine.

No conflict of interest.

